# A Systematic Review of Long Noncoding RNAs in Hepatocellular Carcinoma: Molecular Mechanism and Clinical Implications

**DOI:** 10.1155/2018/8126208

**Published:** 2018-07-09

**Authors:** Xiaoge Hu, Jiahong Jiang, Qiuran Xu, Chao Ni, Liu Yang, Dongsheng Huang

**Affiliations:** ^1^Key Laboratory of Tumor Molecular Diagnosis and Individualized Medicine of Zhejiang Province, Zhejiang Provincial People's Hospital, People's Hospital of Hangzhou Medical College, Hangzhou, Zhejiang 310014, China; ^2^Key Laboratory of Gastroenterology of Zhejiang Province, Zhejiang Provincial People's Hospital, People's Hospital of Hangzhou Medical College, Hangzhou, Zhejiang 310014, China; ^3^Department of Second Clinical Medical College, Zhejiang Chinese Medicine University, Hangzhou, Zhejiang 310053, China; ^4^Department of General Surgery, Zhejiang Provincial People's Hospital, People's Hospital of Hangzhou Medical College, Hangzhou, Zhejiang 310014, China

## Abstract

Hepatocellular carcinoma (HCC) has the second highest mortality rate worldwide among all cancers. Previous studies have revealed the significant involvement of long noncoding RNAs (lncRNAs) in numerous human cancers including HCC. Both oncogenic and tumor repressive lncRNAs have been identified and implicated in the complex process of hepatocarcinogenesis. They can be further explored as prospective diagnostic, prognostic, and therapeutic markers for HCC. An in-depth understanding of lncRNAs' mechanism in HCC is therefore required to fully explore their potential role. In the current review, we will concentrate on the underlying function, molecular mechanisms, and potential clinical implications of lncRNA in HCC.

## 1. Introduction

Among all cancers, hepatocellular carcinoma (HCC) has the second highest mortality rate worldwide [1]. The risk factors, including HBV or HCV infection, alcoholism, liver cirrhosis, and metabolic diseases, contribute to HCC [2]. The molecular mechanism of hepatocarcinogenesis is highly complex and involves an interplay between dysregulated cell cycle, apoptosis, tumor cell invasion, and metastasis [2]. Despite advances in diagnosis and therapy, the incidence and mortality of liver cancer continue to increase [3]. It is vital therefore to illustrate the molecular mechanism of HCC in order to improve diagnosis, treatment, and overall prognosis.

With the development of human genome sequencing technology, about 20000 protein-coding genes have been identified, which account for less than 2% of the entire genome [4]. In fact, greater than 90% of the human DNA would be converted into noncoding RNAs (ncRNAs), which, despite not being translated into proteins, are involved in several cellular functions [5, 6]. The long ncRNAs (lncRNAs) with more than 200 nucleotides play significant roles in cell growth and differentiation, chromatin organization, and regulation of gene expression [7, 8]. lncRNAs are classified into intronic, intergenic, sense, and antisense types based on their genomic location [9] and into signaling, decoy, guide, and scaffold lncRNAs on a functional basis [10]. Signaling lncRNAs mainly act as transcription factors or as intermediates in various signaling pathways [10], and decoy lncRNAs act as “molecular sponges” by binding to and sequestering transcription factors away from their target genes [10]. Guide lncRNAs can regulate gene expression through chromatin remodeling by recruiting chromatin-modifying enzymes [10]. Finally, the scaffold lncRNAs act as recruiting platforms for multiple proteins and form lncRNA-ribonucleoprotein (lncRNA-RNP) complexes, which subsequently regulate downstream signaling [10].

Numerous lncRNAs have been identified recently with the help of high-throughput sequencing and microarrays. Most of them are aberrantly expressed in tumors like HCC, breast cancer, lung cancer, colorectal cancer, and others [11]. lncRNAs are known to regulate cell proliferation, epithelial-mesenchymal transition (EMT), angiogenesis, metastasis, autophagy, and so forth. Considering their cancer specific expression and detectable presence in clinical samples like blood and urine, lncRNAs are potential diagnostic markers for tumors. Therefore, a better understanding of HCC specific lncRNAs will greatly contribute to the diagnosis and treatment of HCC.

lncRNAs exhibit both tumor suppressive and oncogenic roles. In the present review, we will concentrate mainly on the functions, molecular mechanisms, and potential clinical implications of HCC-related lncRNAs that are abnormally expressed and therefore have critical roles in hepatocarcinogenesis.

## 2. Upregulated/Oncogenic lncRNAs in HCC

### 2.1. HULC

“Highly upregulated in liver cancer” or HULC, a 500 bp lncRNA, was the earliest lncRNA reported to be highly expressed in HCC [12]. In addition to the tumor tissues, significantly greater levels of HULC were also found in HCC cell lines and plasma of patients [12–16], indicating its potential role as a biomarker of HCC. HULC is involved in multiple cellular processes like proliferation, EMT, angiogenesis, autophagy, and chemoresistance (Table 1). Furthermore, HULC overexpression was linked with tumor size [17], clinical TNM stage [16], and recurrence and overall survival (OS) in HCC [18].

Wang et al. reported a decoy role of HULC wherein it downregulated miR-327 by its molecular sponge function [13]. HULC-induced miR-327 inhibition lifted the miR-327-mediated translational suppression of PRKACB, which consecutively activated the cAMP response element binding protein (CREB) [13]. CREB induced expression of HULC, thereby forming a CREB-HULC-PRKACB positive feedback loop [13]. HULC also acted as a molecular decoy to downregulate miR-186 which upregulated HMGA2 and lead to HCC progression. In this model, HULC expression was regulated by IGF2BP1 by accelerating HULC degradation [16]. Various studies have elucidated the pathways through which HULC promotes hepatocarcinogenesis: it activates angiogenesis via the HULC/miR-107/E2F1/SPHK1 axis [19], enhances EMT and metastasis via the HULC/miR-200a-3p/ZEB1 axis [18], induces autophagy via the HULC/USP22/Sirt1 axis [20], and augments cell proliferation by stabilizing COX-2 [21] (Table 2). HULC is also involved in hepatitis B virus (HBV) induced HCC, in which HBx plays an important role [22]. HBx markedly increased cell proliferation by upregulating HULC and inhibiting p18, while HULC inhibition abolished HBx-induced cell proliferation accompanied by p18 upregulation [14]. Taken together, HULC is a potential biomarker for diagnosing HCC.

### 2.2. HOTAIR

“HOX transcript antisense intergenic RNA” or HOTAIR is a lncRNA (2.2 kb length) which originates from the* HOXC* antisense strand [23]. HOTAIR is overexpressed in HCC cells and tissues [24–27] and is associated with worse prognosis, shorter recurrence-free survival, and increased risk of recurrence after hepatic transplantation [26, 28, 29]. Functionally, HOTAIR enhances proliferation, migration, glycolysis, autophagy, and chemoresistance in HCC cells (Table 1).

HOTAIR-mediated inhibition of miRNA-218 induced Bmi-1 expression and activated downstream P14 and P16 signaling, contributing to hepatocarcinogenesis [25]. FOXC1 upregulated HOTAIR in HCC cells via miR-1 inhibition, thereby increasing proliferation [30]. In addition, HOTAIR also increased cell proliferation by regulating OGFr [31]. HOTAIR silencing in Huh7 cells decreased proliferation and induced cisplatin resistance via inhibition of STAT3 and ABCB1, which was rescued by inhibiting STAT3 phosphorylation [32] (Table 1). Wei et al. showed that HOTAIR-induced upregulation of GLUT1 and activation of mTOR signaling pathway facilitate glycolysis in HCC cells [27], indicating a direct association between HOTAIR and glucose metabolism in cancer cells. RNAi-mediated HOTAIR knockdown in HCC cells upregulated the RNA binding motif protein 38 (RBM38) [33] (Table 1). Furthermore, knockdown of RBM38 could restore HOTAIR-knockdown-induced decrease in cell migration and invasion [33]. Thus, HOTAIR likely enables HCC metastasis and invasion by inhibiting RBM38. The PRC2 complex, consisting of SUZ12 and EZH2, plays a key role in hepatocarcinogenesis [34–36]. HOTAIR also acts as a scaffold by recruiting PRC2 to the LSD1/Co-REST/HDAC1 complex [37]. In addition, HOTAIR also promotes HBV-mediated HCC by accelerating the degradation of SUZ12 and ZNF198 [38] (Table 1). Finally, HOTAIR could also induce autophagy in HCC cells by upregulating ATG3 and ATG7 [39] (Table 1). Taken together, HOTAIR promotes hepatocarcinogenesis by multiple mechanisms.

### 2.3. MALAT1

Overexpressed “metastasis-associated lung adenocarcinoma transcript 1” or MALAT1 has been initially discovered in human non-small-cell lung cancer (NSCLC) [40]. MALAT1 is overexpressed in HCC tissues and cell lines [41, 42] and is linked with a higher tumor recurrence rate in patients after hepatic transplantation, indicating a predictive role of MALAT1 in HCC recurrence [42]. Functionally, MALAT1 promotes proliferation, invasion, metastasis, chemosensitivity, and autophagy in HCC cells (Table 1).

MALAT1 is upregulated by Sp1 and Sp3 and downregulated by MIT (Sp1 binding inhibitor), indicating a possibility of targeting MALAT1 in HCC patients by MIT [43]. High expression of MALAT1 is linked with 5-FU resistance in HCC cell line [44]. In addition, HIF-2*α* inhibits miR-216b through MALAT1, where the HIF-2*α*-MALAT1-miR-216b axis promotes autophagy with LC3-II upregulation and p62 downregulation, contributing to HCC chemosensitivity [44] (Table 1). MALAT1 also promotes arsenite-induced glycolysis via stabilizing HIF-1*α* in human hepatic L-02 cells [45]. Moreover, MALAT1, negatively regulated by p53, enhanced proliferation during liver regeneration through stimulation of the Wnt/*β*-catenin pathway [46] (Table 1). The mTOR signaling pathway is essential for the oncogenic role of MALAT1, which further mediates SRSF1 upregulation and mTOR activation [47]. MALATI promotes tumor growth, invasion, and metastasis of HCC as a decoy lncRNA through the MALAT1/miR-143-3p/ZEB1 axis and inhibiting miR-146b-5p [48, 49]. It can also act as a ceRNA for miR-195 and reverse miR-195-mediated EGFR inhibition and further promote cell proliferation by activating the PI3K/AKT and JAK/STAT pathways, indicating a role of MALAT1-miR-195-EGFR axis in HCC [50] (Table 1). Like HULC and HOTAIR, MALAT1 is also involved in HBx-mediated hepatocarcinogenesis [51]; it is upregulated by HBx and enhances proliferation and metastasis by activating LTBP3 [51], forming the HBx-MALAT1-LTBP3 axis. Taken together, MALAT1 regulates multiple cellular processes through its decoy or ceRNA functions, indicating a potential target for HCC therapy.

### 2.4. HOTTIP

“HOXA transcript at the distal tip” or HOTTIP is greatly expressed in HCC tumor tissues and cells [52] and is linked with a greater threat of metastasis and poor OS [52]. Studies have shown the effect of HOTTIP on HCC proliferation, metastasis, and glutamine metabolism (Table 1) [52–54].

HOTTIP downregulates miR-125b, miR-192, and miR-204 and enhances the cell growth and migration through the miR-192/-204-HOTTIP axis [53, 54], while HOTTIP inhibition decreases growth of HCC cells [52, 54]. In addition, HOXA13 and GLS1 are further revealed to be the likely target genes of miR-192/-204-HOTTIP axis, and overexpression of miR-192 and miR-204 is associated with increased survival in the patients [54]. Taken together, these findings imply the oncogenic role of HOTTIP in hepatocarcinogenesis through miRNA interaction.

### 2.5. MVIH

“Microvascular invasion in HCC” or MVIH is situated at chromosome 10 and was firstly identified by Yuan et al. in HCC [55]. High levels of MVIH in HCC were correlated with enhanced invasion and poor prognosis with decreased RFS and OS [55]. As shown in Table 1, MVIH plays important roles in proliferation, migration, apoptosis, metastasis, and angiogenesis in HCC [55–57].

MVIH exerts its proangiogenic action by inhibiting PGK1 secretion [55]. It also acts like a sponge for miR-199, and MVIH-mediated inhibition of miR-199 leads to increased proliferation and apoptosis inhibition in HCC cells [56]. Recently, MVIH was reported to control proliferation and migration of HCC cells via modulation of ARID1A-mediated regulation of CDKN1A [57]. Taken together, these findings underscore the oncogenic role of MVIH in HCC.

### 2.6. PVT1

Murine PVT1 was first identified in the liver where it accelerated proliferation and cell cycling and enhanced stem-cell-associated properties [58]. Human PVT1 is overexpressed in HCC tumor tissues and cell lines and is linked with advanced TNM stage and poor prognosis as well as RFS [58–60], and upregulation of PVT1 can also predict HCC recurrence [59]. As shown in Table 1, PVT1 plays an oncogenic role in multiple cellular processes like proliferation and invasion and increases the stemness of HCC cells [58, 60, 61].

Functionally, through interaction between PVT1 and NOP2, PVT1 enhances the expression of NOP2 via stabilizing NOP2, thus promoting proliferation, cell cycle, and stemness of HCC cells [58] (Table 1). In addition, PVT1 can also induce miR-214 inhibition via interaction with EZH2 to promote cell proliferation and invasion [60], forming a PVT1/EZH2/miR-214 axis (Table 1) [60]. The clearly oncogenic role of PVT1 indicates its potential use as a biomarker in diagnosing and predicting recurrence in HCC.

In addition to the lncRNAs mentioned above, several others are upregulated during hepatocarcinogenesis, including DANCR, HEIH, and Linc-ROR (Table 1).

## 3. Downregulated/Tumor Suppressive lncRNAs in HCC

### 3.1. H19

H19 is situated on chromosome 11p15.5 [62] and plays a key role in various cancers including HCC, where the abnormal expression of H19 is linked with late stages of cancer and poor DSF and outcome [63–65]. Functionally, H19 regulates proliferation, migration, invasion, EMT, metastasis, and chemoresistance in HCC cells [64, 66–68] (Table 2).

H19 is upregulated in doxorubicin-resistant R-HepG2 cells [66] and induces drug resistance by modulating MDR1 [66]. H19 overexpression enhanced the tumor growth in* in vivo* models of HCC, while H19 inhibition decreased [67]. HCC patients with elevated expression of H19 in the tumor tissues showed poor DFS, suggesting a predictive role of H19 in HCC prognosis [65]. However, some studies have shown H19 to be significantly downregulated in HCC [64, 65], which is correlated with poor prognosis [64]. In addition, H19 could also activate miR-200 and suppress tumor metastasis and EMT [64] (Table 2). H19 inhibition by miR-675 promoted metastasis of HCC via the AKT/GSK-3beta/Cdc25A pathway [68] (Table 2). Taken together, H19 seems to act as a tumor suppressor as well as an oncogene in HCC.

### 3.2. MEG3

“Maternally expressed 3” or MEG3 is a maternally inherited lncRNA presented on chromosome 14q32.3 [69] and was first identified by Miyoshi et al. [70]. MEG3 expression is reportedly low in human HCC cells [71–73] and is linked with reduced OS, suggesting a predictive role of MEG3 in HCC prognosis [73]. As shown in Table 2, MEG3 could regulate proliferation and apoptosis in HCC cells [72–75].

MEG3 can be negatively regulated by UHRF1 via modulating DNA methylation, since its promoter region is highly methylated [73]. One mechanism of MEG3 mediated tumor suppression is the activation of p53 by increasing its stability and modulating the downstream genes [72, 74] (Table 2). Using a novel delivery system, MEG3 was introduced into HCC cells and resulted in tumor growth inhibition via the p53 signaling, indicating a bona fide tumor suppressive role of MEG3 in HCC [74]. Furthermore, it acted as a molecular sponge for miR-664 and could inhibit cell proliferation by modulating miR-664-mediated regulation of ADH4 [76] (Table 2). Taken together, MEG3 is a tumor suppressor and might be considered a prospective diagnostic, predictive and therapeutic biomarker in HCC.

### 3.3. Dreh

“Downregulated expression by HBx” or Dreh was first identified by lncRNA microarray on WT and HBx-transgenic mice [77]. It is low expressed in the tumor tissues of HBV-related HCC patients and corresponding cell lines [77, 78]. Patients with decreased expression of Dreh showed poor survival [77]. As shown in Table 2, Dreh is linked with the proliferation and metastasis of HBV-related HCC.

A previous study revealed a negative correlation of Dreh expression with HBx and HBs [78]. Dreh is downregulated by HBx via downregulation of vimentin, which results in the suppression of HCC growth and migration [77, 78] (Table 2), thus underscoring the tumor suppressive role of Dreh in HBV-related HCC.

### 3.4. LET

“Low expression in tumor” or LET is present in significantly low levels in HCC tumor tissues [79] and is linked with metastasis [79]. As shown in Table 2, LET influences the invasiveness and metastasis of HCC cells.

LET is downregulated by HDAC3 [79], and LET inhibition increases the stability of NF90, thus promoting hypoxia-induced invasion [79] (Table 2). This was successfully validated in an HCC clinical sample with abnormal histone acetylation, downregulation of LET, and upregulation of NF90. These findings suggest a tumor suppressive role of LET centered around regulating metastasis under hypoxia.

As shown in Table 2, along with the lncRNAs discussed above, several others have been indicated to influence hepatocarcinogenesis, such as ZNFX1-AS1, PTENP1, and XIST.

## 4. lncRNAs as Diagnostic Biomarkers and Drug Targets in HCC

Increasing evidence shows critical roles of various lncRNAs in hepatocarcinogenesis, either as tumor suppressors or as oncogenes. Abnormal expression of lncRNAs is significantly linked with cancer proliferation, metastasis, OS, DFS, RFS, and the tumor TNM stage. Multivariate analyses have further revealed that lncRNAs can independently predict recurrence and outcomes of HCC. With the rapid development of molecular diagnostics such as sequencing technology, qRT-PCR, microarrays, and RNA immunoprecipitation, lncRNAs can be easily detected in various body fluids, thus paving the way for lncRNA as novel diagnostic and prognostic markers of HCC. For example, the oncogenic HULC is significantly upregulated in plasma of patients as well the HCC tumor tissues; thus, it could serve as a novel diagnostic biomarker for HCC (Table 3) [15, 17]. In addition to plasma, serum and exosomes can also be used for lncRNA detection. For example, HEIH, an oncogenic lncRNA expressed highly in HCC tissues, was also found to be overexpressed in the serum and exosomes of patients with HCV-related HCC (Table 3). In addition to HULC and HEIH, many other lncRNAs could also serve as biomarkers of HCC which are shown in Table 3.

Since various lncRNAs are abnormally expressed in HCC and affect many downstream genes and related signaling pathways through oncogenic or tumor suppressive action, restoring these lncRNAs to their normal expression level is a therapeutic option worth considering, especially as an alternative to the chemotherapeutic drugs which usually result in chemoresistance [80]. Pharmaceutical companies have recently shown a great interest in lncRNA-targeted therapy and have already taken actions [81, 82]. lncRNAs could be upregulated by exogenous overexpression and directly targeted by their specific siRNAs or antisense oligonucleotides [83, 84]. For example, the tumor suppressor MEG3 introduced into HCC tumor through a novel delivery system effectively induced apoptosis in HCC cells [74], presenting a potential lncRNA-targeted therapy with fewer side effects. Therefore, clarifying the specific mechanism of lncRNA action will greatly promote the advancement of lncRNA-based diagnosis and therapy for HCC.

## Figures and Tables

**Table 1:** Mechanisms and biological functions of upregulated LncRNAs in HCC.

lncRNA	Full name	Mechanism	Function	References
HULC	Highly upregulated in liver cancer	Downregulating miR-372 and miR-186; downregulating p18, SPHK1, and ZEB1; activating USP22/COX-2 axis and USP22/Sirt1 axis; upregulating HMGA2	Proliferation(+), EMT(+), angiogenesis(+), metastasis(+), autophagy(+), chemoresistance(+)	[13, 14, 18–21, 85, 86]

HOTAIR	HOX transcript antisense RNA	Downregulating RBM38, miR-1, miRNA-218, SETD2, SUZ12, and ZNF198; activating P14, P16, GLUT1, MMP9, VEGF, ATG3, and ATG7	Proliferation(+), migration(+), invasion(+), glucose metabolism(+), autophagy(+)	[25–30, 33, 38, 39, 87]

MALAT1	Metastasis-associated lung adenocarcinoma transcript 1	HIF-2*α*-MALAT1-miR-216b axis; MALAT1/miR-143-3p/ZEB1 axis; MALAT1-miR-195-EGFR axis; HBx-MALAT1/LTBP3 axis; upregulating HIF-1*α*, Wnt/*β*-catenin pathway, SRSF1, and mTOR pathway	Proliferation(+), migration(+), invasion(+), chemoresistance(+), autophagy(+), metastasis(+)	[42, 44–51]

HOTTIP	HOXA transcript at the distal tip	miR-125b/HOTTIP axis; miR-192/-204-HOTTIP axis	Metastasis(+), proliferation(+)	[52–54]

MVIH	Microvascular invasion in HCC	Downregulating miR-199a	Angiogenesis(+)	[55, 56]

PVT1	Plasmacytoma variant translocation 1	PVT1/NOP2 axis; PVT1/EZH2/miR-214 axis	Proliferation(+), cancer cell stemness(+)	[58–61]

UCA1	Urothelial carcinoma associated-1	UCA1/miR-203/Snail2 axis; HBx-UCA1/EZH2-p27Kip1 axis; UCA1-miR-216b-FGFR1-ERK axis	Proliferation(+), invasion(+), EMT(+)	[88–91]

ATB	Activated by TGF-*β*	ATB/miR-200/ZEB1-ZEB2 axis	EMT(+), invasion(+), metastasis(+)	[92–94]

Linc-ROR	LincRNA regulator of reprogramming	Downregulating miR-145–HIF-1*α*	Chemoresistance(+), EMT(+), invasion(+), metastasis(+)	[95–97]

VLDLR	Very low density lipoprotein receptor	Downregulating ABCG2	Chemoresistance(+)	[98]

CCAT1	Colon cancer associated transcript 1	Downregulating let-7	Proliferation(+), migration(+), invasion(+)	[99]

Linc00974	Long intergenic non-protein-coding RNA 974	Upregulating KRT19, Notch, and TGF-*β* signaling	Proliferation(+), metastasis(+)	[100]

HNF1A-AS1	HNF1A antisense RNA 1	HNF1A-AS1-miR-30b axis; downregulating NKD1 and p21	Apoptosis(−), autophagy(+), proliferation(+)	[101, 102]

HEIH	Highly expressed in HCC	Upregulating EZH2	Proliferation(+), invasion(+)	[85, 103]

HBx-LINE1	Fusion of the human cellular long interspersed nuclear elements and HBx	Downregulating miR-122; upregulating Wnt signaling	EMT(+), invasion(+), metastasis(+)	[104, 105]

LncTCF7 (WSPAR)	WNT signaling pathway activating noncoding RNA	Upregulating Wnt signaling	EMT(+), invasion(+), metastasis(+), cancer stem cell self-renewal(+)	[106]

DANCR	Differentiation antagonizing non-protein-coding RNA	Downregulating CTNNB1	Cancer cell stemness(+)	[107, 108]

BANCR	BRAF-regulated lncRNA 1	Activating MEK	Invasion(+), metastasis(+)	[109]

ZEB1-AS1	ZEB1 antisense RNA 1	Upregulating ZEB1	EMT(+), invasion(+), metastasis(+), proliferation(+)	[110]

DBH-AS1	DBH antisense RNA 1	Activating MAPK signaling; upregulation of CDK6, CCND1, and CCNE1; downregulating p16, p21, and p27	Proliferation(+)	[111]

TUC338	Transcribed ncRNA encoding uc.338	TUC338/RASAL1 axis	Proliferation(+); chemoresistance(−)	[112]

TUG1	Taurine upregulated 1	TUG1-miR132-Hedgehog axis; TUG1/miR-455-3p/AMPK*β*2 axis	Proliferation(+), apoptosis(−), metastasis(+)	[113, 114]

ANRIL (CDKN2B-AS1)	CDKN2B antisense RNA 1	Downregulating miR-122-5p	Proliferation(+), apoptosis(−), metastasis(+)	[115, 116]

URHC	Upregulated in hepatocellular carcinoma	Downregulating ZAK; suppressing ERK/MAPK pathway	Proliferation(+), apoptosis(−)	[117]

AFAP1-AS1	AFAP antisense RNA 1	Upregulation of RhoA/Rac2 signaling	Proliferation(+), invasion(+)	[118]

PCNA-AS1	PCNA antisense RNA 1	Stabilizing PCNA	Proliferation(+)	[119]

CCAT2	Colon cancer associated transcript 2	Upregulating FOXM1 expression;	Proliferation(+), apoptosis(−), EMT(+)	[120–122]

SNHG1	Small nucleolar RNA host gene 1	Downregulating miR-195	Proliferation(+), invasion(+), migration(+), apoptosis(+)	[123, 124]

HCAL	HCC-associated lncRNA	HCAL-miR-15a/miR-196a/miR-196b-LAPTM4B network	Proliferation(+), metastasis(+)	[125]

MUF	MSC-upregulated factor	Activating Wnt/*β*-catenin signaling	EMT(+)	[126]

HOXD-AS1	HOXD cluster antisense RNA 1	Upregulating SOX4, EZH2, and MMP2; HOXD-AS1/miR19a/ARHGAP11A axis	Migration(+), invasion(+), apoptosis(−)	[127, 128]

AWPPH	None	Activating PIK3CA	Proliferation(+), migration(+)	[129]

SNHG12	Small nucleolar RNA host gene 12	SNHG12/miR-199a/b-5p/MLK3 axis	Proliferation(+), invasion(+), metastasis(+), apoptosis(−)	[130]

lncBRM	lncRNA for association with Brahma	Activating YAP1 signaling	Cancer stem cell self-renewal(+)	[131]

Unigene56159	None	Unigene56159/miR-140-5p/Slug axis	EMT(+), migration(+), invasion(+)	[132]

SNHG6-003	None	Sponge for miR-26a/b	Proliferation(+), chemoresistance(+)	[133]

lnc-*β*-Catm	None	Activating Wnt-*β*-catenin signaling	Proliferation(+), cancer stem cell self-renewal(+)	[134]

+: increase; −: decrease.

**Table 2:** Mechanism and biological function of downregulated LncRNAs in HCC.

lncRNA	Full name	Mechanism	Function	Reference
H19	None	Activating miR-200 family, downregulating AKT/GSK-3beta/Cdc25A pathway	Migration(−), invasion(−), metastasis(−), EMT(−)	[64, 68]

MEG3	Maternally expressed gene 3	Activating P53, MEG3/miR664/ADH4 axis	Proliferation(−), apoptosis(+),	[72, 73, 76]

Dreh	Downregulated by HBx	Downregulating vimentin	Proliferation(−), migration(−), metastasis(−)	[77]

LET	Low expression in tumor	Downregulating NF90	Invasion(−), metastasis(−)	[79]

ZNFX1-AS1	ZNFX1 antisense RNA 1	Upregulating miR-9	Proliferation(−), apoptosis(+),	[135]

PTENP1	Phosphatase and tensin homolog, pseudogene 1	Downregulating miR-17, miR-19b, and miR-20a	Proliferation(−), angiogenesis(−), apoptosis(+), autophagy(+)	[136]

AOC4P	Amine oxidase, copper containing 4, pseudogene	Downregulating vimentin	Proliferation(−), migration(−), invasion(−), EMT(−)	[137]

FTX	None	Downregulating miR-374a and Wnt/*β*-catenin signaling	Proliferation(−), invasion(−), metastasis(−), EMT(−),	[138]

XIST	X inactive specific transcript	XIST/miR-181a/PTEN axis; XIST/miR-92b/Smad7 axis	Proliferation(−), invasion(−), metastasis(−)	[139, 140]

LncRNA00364	None	LncRNA00364/STAT3/IFIT2 axis	Proliferation(−), apoptosis(+), G1/S cell cycle progression(−),	[141].

Linc-USP16	None	ceRNA for miR-21 and miR-590-5p and upregulating PTEN	Proliferation(−), migration(−)	[142]

CASC2	Cancer susceptibility candidate 2	Downregulating miR-24-3p, CASC2/miR-367/FBXW7 axis	Proliferation(−), apoptosis(+), migration(−), invasion(−), EMT(−)	[143, 144]

LINC00657	None	LINC00657/miR-106a-5p /PTEN axis	Proliferation(−), migration(−), invasion(−)	[145].

FER1L4	Fer-1-like protein 4	Downregulating miR-106-5p	Proliferation(−), migration(−), invasion(−), apoptosis(+)	[146]

uc.134	None	uc.134/CUL4A/LATS1 axis	Proliferation(−), invasion(−), metastasis(−)	[75]

lnc-DILC	lncRNA downregulated in liver cancer stem cells	IL-6/STAT3 axis	Proliferation(−)	[147]

+: increase; −: decrease.

**Table 3:** LncRNAs as biomarkers in HCC.

lncRNA	Expression in HCC	Potential implications	Sample	References
HULC	Up	Detection, metastasis, prognosis,	Plasma	[15, 17]
Linc00152	Up	Detection, metastasis	Plasma	[17, 148]
uc001ncr	Up	Detection, HBV-related HCC	Serum	[149]
AX800134	Up	Detection, HBV-related HCC	Serum	[149]
PVT1	Up	Detection	Serum	[150]
uc002mbe.2	Down	Detection	Serum	[150]
RP11-160H22.5	Up	Tumorigenesis	Plasma	[148, 151]
XLOC014172	Up	Tumorigenesis, metastasis	Plasma	[148, 151]
LOC149086	Up	Tumorigenesis, metastasis	Plasma	[151]
HEIH	Up	Detection, HCV-related HCC	Serum, exosomes	[103]
UCA1	Up	Detection, prognosis	Serum	[88]
DANCR	Up	Detection	Plasma	[108]
lncRNA-CTBP	Up	Detection	Serum	[152]
Linc00974	Up	Detection, metastasis	Plasma	[100]
